# How Important Is the Etiology in the Treatment of Epiphora?

**DOI:** 10.1155/2016/1438376

**Published:** 2016-08-10

**Authors:** Mahmut Oğuz Ulusoy, Sertaç Argun Kıvanç, Mehmet Atakan, Berna Akova-Budak

**Affiliations:** ^1^Department of Ophthalmology, Başkent University Konya Research Hospital, 42000 Konya, Turkey; ^2^Department of Ophthalmology, Uludağ University School of Medicine, 16059 Bursa, Turkey; ^3^Department of Ophthalmology, Aksaray State Hospital, 68000 Aksaray, Turkey

## Abstract

*Purpose.* There are several etiological factors that cause epiphora, and treatment differs according to the cause. We aimed to evaluate the etiology of epiphora and the treatment modalities of the affected patients.* Materials and Methods.* Data of patients who were referred to ophthalmology clinics for epiphora were retrospectively analyzed. All patients were evaluated for epiphora etiology, treatment modalities, and duration of complaints, after complete ophthalmologic examination.* Results.* This study consisted of 163 patients with a mean age of 64.61 ± 16.52 years (range 1–92 years). Lacrimal system disease (48.4% [79/163]) was the most common cause, followed by ocular surface disease (dry eye/blepharitis) (38.7% [63/163]). Among the patients included in this study, 69% (113/163) did not receive any treatment, whereas only 1.8% (3/163) were treated surgically. About 4.3% of the patients (7/163) had a complaint for more than 5 years (*p* = 0.012) and six of these had chronic dacryocystitis and one had ectropion.* Conclusion.* Epiphora not only has a negative impact on patients' comfort, but also puts them at risk for probable intraocular operations in the future. Therefore, the wide range of its etiology must be taken into consideration and adequate etiology-specific treatment options must be applied.

## 1. Introduction

Epiphora is one of the most common complaints of patients consulting ophthalmology clinics. It causes much discomfort for patients in their daily lives and also puts the safety of several intraocular surgeries and surgical attempts at risk [1].

The primary cause of epiphora is an obstruction that prevents the drainage at any level of the nasolacrimal system or even the lack of drainage related to eyelid diseases. Another cause may be the reflex hypersecretion related to ocular surface diseases such as dry eye or the combination of all these situations [2]. Due to the various possible etiological factors, different approaches for different situations are needed. However, it is observed that cases requiring surgical intervention are delayed due to patients' fear of surgery, nonrecommendation of surgery by doctors or negative view points of the society, and other similar reasons; therefore, the disease eventually becomes chronic.

In this study, we aimed to retrospectively investigate the etiologies, duration, and treatment modalities for epiphora in patients who consulted our clinic about eye watering complaints.

## 2. Materials and Methods

A retrospective analysis of the records of patients who consulted our clinic about eye watering complaints from June 2013 to January 2014 was conducted. The following data were collected from the patients: duration of complaints, whether they were previously treated, and the treatments they received.

The patients included in our study were divided into four primary categories based on the etiology: (1) ocular surface diseases (dry eye, blepharitis, etc.), (2) lacrimal system diseases (chronic dacryocystitis, congenital dacryostenosis, punctal stenosis, etc.), (3) eyelid anomalies (entropion, ectropion, etc.), and (4) others. All the patients underwent complete ophthalmologic examination. Lid position anomalies were evaluated; entropion, ectropion and the accompanying punctal stenosis, and additional pathologies were recorded. Punctual openings, eyelash anomalies (trichiasis and distichiasis), blepharitis, and presence of additional problems were investigated by biomicroscopic examination. The diagnosis related to dry eye was made based on the presence of at least two of the following symptoms: hypersecretion, presence of corneal fluorescein staining, tear break-up time ≤ 10-s, and Schirmer's test performed under anesthesia under 10 mm. Children examined for congenital dacryostenosis were asked to wait for 5 min after the administration of eye drops containing fluorescein and the diagnosis was made after the fluorescein disappearance test. Patients who were suspected of having chronic dacryocystitis underwent nasolacrimal duct irrigation and the diagnosis was confirmed by contrast dacryocystography. In addition to the basic above-mentioned diagnoses, punctum anomalies such as the mass closing off the punctum, conjunctivochalasis, inflamed pterygium, and double punctum, likely to cause epiphora, were grouped separately as other causes of epiphora.

Patients were excluded if they had a concurrent ocular infection, a history of facial palsy, ocular or periocular trauma, facial radiotherapy, and ocular medication except that given for epiphora.

This study adhered to the tenets of the Declaration of Helsinki to review the patients' data.

Data were analyzed using SPSS 21 (SPSS, Inc., Chicago, IL, USA). The correlation among the variables was evaluated using Pearson's chi-square test. The group averages were compared using the one way analysis of variance test and post hoc evaluation was performed using Tukey's test. A *p* value <0.05 was considered statistically significant.

## 3. Results

The average age of the 163 patients included in this study was 64.61 ± 16.52 years (range: 1–92 years), and 52.1% (85/163) were females. The duration of complaints ranged from 1 month to 1 year in 49.7% of patients (81/163), from 1 to 5 years in 41.1% of patients (67/163), and ≥5 years in 4.3% (7/163) of patients.  Table 1 shows the causes of epiphora, the duration of complaints, and the relationship with gender. The chief complaint in 35.6% (58/163) of the patients was related to only one eye, and it was bilateral in the remaining patients. Bilaterality was significantly higher in men than in women (*p* = 0.016) and dry eye was more bilateral than other pathologies (*p* < 0.001). Half of the patients had one-eye complaints in the right eye and the other half in the left eye. While the pathologies were different in each eye in 3.8% (4/105) of the patients with bilateral complaints, they were similar in others. In 21 patients (12.8%), there was a combination of more than one pathology in one eye. These combinations were as follows: three of them had a combination of chronic dacryocystitis and punctum stenosis, two of them had a combination of ectropion and chronic dacryocystitis, and the remaining 16 had a combination of ectropion and punctum stenosis.

When the epiphora groups were examined, lacrimal system diseases were present in 48.4% (79/163) of the patients. But when we considered the diseases individually, ocular surface disease (dry eye/blepharitis) was the topmost with a rate of 38.7% (63/163).  Table 2 shows the causes of epiphora and the relationship with treatment methods and treatment rates.

**Figure 1:**
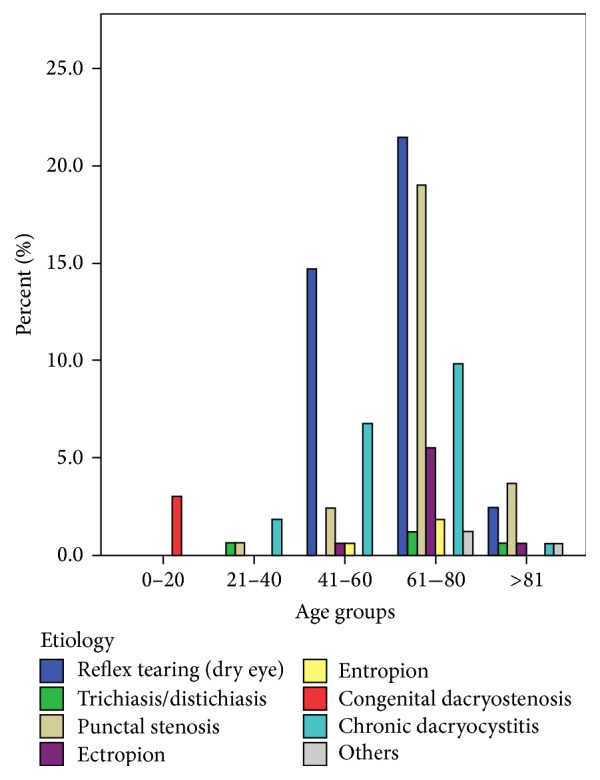
Etiology distribution in different age groups.

Concerning the etiologies, the average age of patients with the pathologies was significantly different between each other (*p* < 0.001). Besides, dry-eye patients were significantly younger than patients who presented with eyelid pathologies (*p* = 0.038). Otherwise, there was no correlation between age and duration of complaints (*p* = 0.105) and no significant difference between the ages of treated and nontreated patients (*p* = 0.073).  Figure 1 depicts the etiology distribution in different age groups.

**Figure 2:**
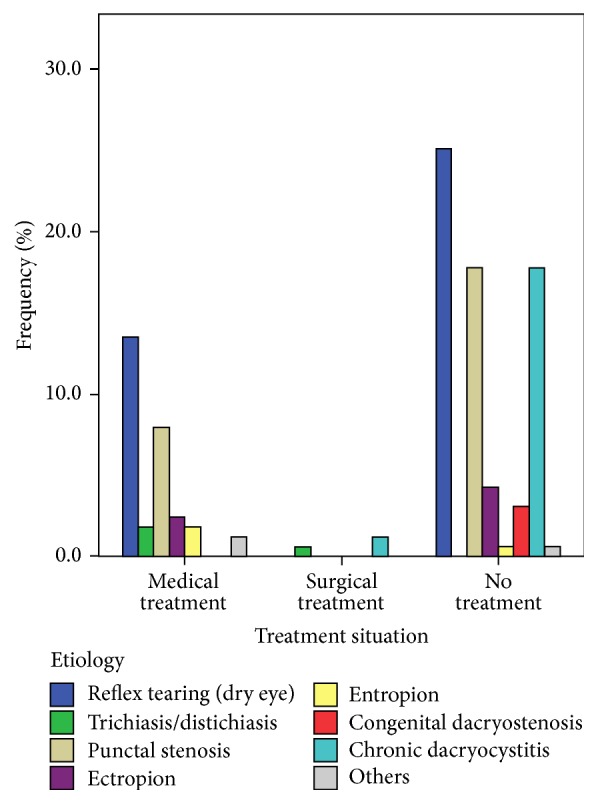
Treatment situation in different etiologies.

While 69.3% (113/163) of the patients included in this study had never received any treatment, 28.8% (47/163) of them had received medical treatment and only 1.8% (3/163) of them had received surgical treatment. Overall, 65.1% (41/63) of the patients with ocular surface disease (dry eye/blepharitis) had never received any treatment, and 70.7% of those without any treatment had complaint periods between 1 month and 1 year: the complaints of 9.7% of them had a duration varying from 1 to 5 years (*p* < 0.05). Only three of the patients who consulted us throughout the study had a previous surgery, two (5.5%, 2/36) of them had chronic dacryocystitis, and the other one (20%, 1/5) suffered from entropion. Treatment modalities of the different etiologies are shown in Figure 2.

## 4. Discussion

Epiphora is one of the most common complaints of patients consulting ophthalmology clinics, which has been described since ancient times in Egyptian papyrus artifacts and during the era of Hippocrates [3]. This disease causes discomfort and problems in the daily activities of individuals, such as reading, daytime and nighttime driving, working on a computer, and watching the television [4]. Beside these functional problems, the cosmetically bad appearance can be annoying, especially in eyelid problems such as ectropion. However, there are numerous etiological factors leading to this complaint.

To the best of our knowledge, there are only few studies in the literature that have investigated the etiology of epiphora. One of the first studies found on this subject was performed by Mainville and Jordan, which revealed that the lacrimal passage obstruction related to epiphora is found in 48.7% of the cases, followed by dry-eye-related reflex tear secretion in 40% [5]. Similarly, in a recent study nasolacrimal duct obstruction (obstruction at any point within the nasolacrimal system) was the most frequent cause of epiphora, with a rate of 33.3% [6]. In contrast, in the study conducted by Bukhari, while punctum stenosis was the primary cause (37.8%), hypersecretion was the second most frequent (27.7%) cause [7]. In a study that claimed that all the patients who were complaining about epiphora did not have just one problem and some of the cases could be multifactorial, the causes of epiphora were reported to be nasolacrimal duct obstruction, dry-eye secondary hypersecretion, and multifactorial epiphora [2]. On the other hand, another recent study showed that lower lid malposition was the primary cause of epiphora (33.3%) [8]. In our study, while 38.7% (63/163) of the patients had ocular surface disease (dry eye/blepharitis), 25.8% (42/163) of them were found to have punctum stenosis.

Generally, when clinicians see patients with epiphora, the first thing that occurs to their mind is lacrimal system diseases. Lacrimal canal is a long passage that starts at eyelid puncta and ends at inferior nasal meatus. Therefore, the obstruction can be seen at different levels of this passage. Although nasolacrimal duct obstruction can be congenital, it can also develop later in a patient's life. Most of the acquired cases may develop as a result of chronic dacryocystitis that emerges after an inflammatory process in the lacrimal sac [9]. In the articles we reviewed, the cases with nasolacrimal duct obstruction were congenital and acquired without any discrepancy, and its frequency was reported to be between 10.1% and 33.3% [2, 6–8]. In our study, we believed that it would be more appropriate to evaluate the congenital and acquired cases separately; consequently, we found that cases of acquired chronic dacryocystitis represented 22.1% (36/163), and congenital cases represented 3.1% (5/163). Thus, the total number of 41 cases out of 163 (25.1%) was similar to that reported in other studies.

Punctum stenosis is known to develop due to chronic blepharitis, eyelid ectropion, side effects caused by topical or systemic medicine use, or just senility [10]. In addition, chronic blepharitis-related inflammation and cicatricial changes, ectropion, and old age-related tissue atrophy have also been implicated in punctum stenosis [11]. Similarly, another study reported punctum stenosis to be significantly higher in women [12]. In studies conducted without taking into account the patients' complaints on eye diseases, the rate of punctum stenosis ranged from 11% to 54.3% [8, 13, 14]; however, almost half of them (41.9%) had epiphora [13]. The rate of punctum stenosis in patients with an epiphora complaint was reported to be 37.8% [14]. In our study, we evaluated only those patients who consulted our polyclinic about a complaint of epiphora and determined the rate of punctum stenosis as 25.8% (42/163). While chronic blepharitis was found to be the cause in 26 (61.9%) of these patients, the remaining had epiphora caused by secondary ectropion. Similarly, 66.6% of the patients were diagnosed with punctum stenosis, and unlike other studies, they were male patients.

Another common cause of epiphora is eyelid disorders. This group includes entropion, ectropion, trichiasis/distichiasis, and other less common problems which were reported by Tse et al. [15]. The authors especially specified the importance of blinking and the factors that effect this mechanism. However, we did not notice any such factor in our patient group. With the outward turning of the lid margin, ectropion causes the punctum to steer away from the bulbus, thus causing obstruction of lacrimal drainage. When this condition becomes chronic, the secondary punctum stenosis is also added up and the complaints may become more noticeable. In entropion, on the other hand, inward turning of the lid margin into the bulbus may cause the eyelashes to create corneal and conjunctival damage, thereby causing epiphora [16]. Due to involution causes, the rates of ectropion and entropion increase with age; in the general population of people aged ≥50 years, the rate of ectropion occurrence was reported to be 3.9% [17]. On the other hand, in patients who consulted about the complaint of epiphora, the occurrence rates of these types of eyelid malpositions varied from 3.5% to 33.3% [2, 7, 8]. Moreover, we found that 16.6% (27/163) of the cases had an ectropion cause, and 3.1% (5/163) had an entropion cause. We also observed that 26 (96.2%) of the ectropion cases and 4 (80%) of the entropion patients were ≥60 years of age. The fact that the rate of eyelid malposition cases in this study was higher than that in other studies may be justified by the fact that the average age of the patients was higher in our study.

The above-mentioned discussion is so far related to the cases in which the lacrimal drainage is obstructed. However, dry-eye related hypersecretion may surpass the rates of all the cases we discussed above. Dry eye and/or chronic blepharitis may affect the corneal and conjunctival neurosensorial receptors and cause reflex hypersecretion of the lacrimal gland [18]. Although rates of 22–29.2% were found in some studies of oculoplastic clinics, increased rate of 40% was also observed in other studies [2, 5–7]. The researchers concluded that, with these high rates, the assumption that epiphora was synonymous of lacrimal system obstruction was dispelled [2]. Our study demonstrated that 38.7% (63/163) of hypersecretion cases were related to epiphora. Besides, if we consider trichiasis and distichiasis also as a cause of reflex hypersecretion, this rate could increase to 42.3% (69/163). Our rate was higher than that in other studies; however, this study was conducted in an ophthalmology clinic of a state hospital and not in an oculoplastic clinic. Other reasons for this higher rate can be the lack of treatment and maybe the lack of compliance to treatment previously given. On the other hand, when we examine the patient's profile in our polyclinics, we would have thought that the number of dry eye and chronic blepharitis patients, in general, would be higher. However, although patients with different complaints consulted our polyclinics, it should be noted that we investigated only epiphora in our study.

In addition to the frequently found pathologies, there are also some pathologies that are rare but can cause epiphora. In our study, such pathologies included mass closing punctum, conjunctivochalasis, inflamed pterygium, and double punctum anomalies, all of which had a 2.4% (4/163) rate. Conjunctivochalasis is an interesting condition that causes epiphora by mechanically displacing the normal tear meniscus and impeding the flow along the eyelid margin toward the punctum [15]. These rare causes varied between 0.7% and 5.1% in other studies [2, 7].

Epiphora became chronic in patients who did not receive any treatment. In one study, the average duration of the complaint was 41.1 months. When the pathologies were examined individually, it was found that the longest duration of 61.9 months was related to eyelid problems and the shortest duration of 28.5 months was related to dry eye [2]. Similarly, in another study, it was reported that 54.6% of the patients had complaints for ≤6 months, and 15.7% of them had it for ≥1 year. It was also observed that the group with the complaint of nasolacrimal duct obstruction had it for a longer period [7]. In our study, 49.7% (81/163) of the patients reported that they had complaints from 1 month to 1 year, 41.1% (67/163) of them had complaints between 1 and 5 years, and 4.3% (7/163) of them had complaints for ≥5 years.

As long as chronic dacryocystitis patients did not undergo surgical intervention, the duration and intensity of complaints may be longer in these patients compared with others as there is no chance of healing. Moreover, every chronic dacryocystitis patient had a risk of infection for possible intraocular surgery. In a recent study, the majority of the microbiologic spectrum of chronic dacryocystitis was Gram (+) which was compatible with the microbiologic spectrum of endophthalmitis, the most feared infection [19, 20]. A study reported that while 15.7% of the patients had complaints for more than 1 year, 50% of the patients with the complaints of nasolacrimal duct obstruction had it for ≥1 year [6]. In our study, on the other hand, 41.6% (15/36) of chronic dacryocystitis patients had complaints for ≤1 year, 41.6% (15/36) of them between 1 and 5 years, and 16.6% (6/36) for ≥5 years. Besides, 88.8% (32/36) of the patients reported that they never received any treatment. Even though we never questioned the reason why they never received any treatment, the general reasons were fear of operation, rumours of low level of success in operations, and lack of knowledge about the treatment because patients are not recommended to undergo the operation. Given the fact that external dacryocystorhinostomy as a classical method of treatment has a success rate of 70–90%, it is thought-provoking that the number of patients not receiving treatment is so high [21].

Eye lid malpositions such as entropion and ectropion also require surgery. However, as we observed in our study, 78.1% of the eyelid patients who had complaints between 1 and 5 years received medical treatment, despite the lack of medical treatment regimen for these situations, and only 1 (2.4%) entropion patient received surgical treatment. The possible reasons why patients with this particular pathology do not receive surgical treatment can be lack of information and guidance given to the patients by their doctors and lack of etiological evaluation of epiphora.

In conclusion, it should be noted that a complaint of epiphora is not only a discomforting pathology for the patient but can also generate situations that may result in permanent blindness. Moreover, given the fact that older patients are natural candidates for various intraocular surgeries, it should be remembered that nontreatment of these types of pathologies may cause serious complications [1]. Therefore, it should be remembered that patients who consult specialists about these complaints have a very large etiological spectrum, their causes should be sought out, patients should be encouraged for cause-oriented treatment, and the necessary treatment should be implemented.

## Figures and Tables

**Table 1:** Causes of epiphora, duration of complaints, and relationship with gender.

	Patients (*n*/%)	Duration of complaints (*n*/%)				Sex (%)	*p* value
		<1 month	1 month–1 year	1–5 years	>5 years		
*(1) Ocular surface disease*	*63 (38.7)*	*8 (4.9*%)	*42 (25.7*%)	*13 (7.9*%)		*F (57.1)*	*0.083*
(a) Dry eye/blepharitis	63 (38.7)	8 (4.9%)	42 (25.7%)	13 (7.9%)		F (57.1**)**	0.083

*(2) Lacrimal pathway diseases*	*79 (48.4)*		*32 (19.6*%)	*42 (25.7*%)	*6 (3.6*%)	*M (52.5)*	*0.663*
(a) Chronic dacryocystitis	36 (22.1)		15 (9.2%)	15 (9.2%)	6 (3.6%)	F (69.4)	0.052
(b) Congenital lacrimal stenosis	5 (3.06)		5 (3.06%)			M (60)	0.042
(c) Punctum stenosis	42 (25.8)		13 (7.9%)	29 (17.7%)		M (66.6)	0.233

*(3) Eyelid disorders*	*35 (21.4)*		*8 (4.9*%)	*26 (15.9*%)	*1 (0.6*%)	*M (54.3)*	*0.075*
(a) Ectropion	27 (16.6)		5 (3.06%)	21 (12.8%)	1 (0.6%)	M (59.2)	0.151
(b) Entropion	5 (3.06)		1 (0.6%)	4 (2.4%)		M (60)	0.044
(c) Trichiasis/distichiasis	6 (3.6)		2 (1.2%)	4 (2.4%)		F (50)	0.083

*(4) Others*	*4 (2.4)*		*2 (1.2*%)	*2 (1.2*%)		*F (50)*	*0.083*

**Table 2:** Causes of epiphora and the relationship with treatment methods and treatment rates.

	Patients (*n*/%)	Treatment modality (med/sur)	Untreated patients (%)	*p* value
*(1) Ocular surface disease*	*63 (38.7)*	Med	65.07	0.000
(a) Dry eye/blepharitis	63 (38.7)	Med	65.07	0.000

*(2) Lacrimal pathway diseases*	*79 (48.4)*			0.000
(a) Chronic dacryocystitis	36 (22.1)	Surg	88.8	0.000
(b) Congenital lacrimal stenosis	5 (3.06)	Surg	100	0.000
(c) Punctum stenosis	42 (25.8)	Surg	69.04	0.000

*(3) Eyelid disorders*	*35 (21.4)*			0.000
(a) Ectropion	27 (16.6)	Surg	70.3	0.000
(b) Entropion	5 (3.06)	Surg	80	0.000
(c) Trichiasis/distichiasis	6 (3.6)	—	—	0.000

*(4) Others*	*4 (2.4)*	Med/Surg	25	0.000
